# 

**Published:** 1915-07-31

**Authors:** 


### CONTENTS.

PAGE

**Professional Secrecy and Public Justice** ... 363

**The Co-operative Holiday System** ... ... 364

**Hospital and Institutional News ...** ... ... 365

A Sanatorium for Munition Workers—Mothers' Rights and Children's Needs—Week-Ends for Infirmary Superintendents—Medical Certificates and Old-Age Pensions—"I Do Not Know"—The Dispensing of Medicine to the Public—A Grave Charge—Fine for an Unqualified "Doctor"—The Insurance Deadlock in Belfast—The Royal Army Medical Corps and Promotion—The Economical Weapon against Tuberculosis—A Hard-Worked Mental Staff—The Problem of Residence—A Doctor as Grave-digger—The Closing of Schools owing to Infectious Disease—Walthamstow Hospital—The Local Government Board and Contracts—A Chronic Insured Patient—A New Departmental Committee on Poor-Law Orders—An Ill-Founded Rumour about a Hospital Ship—Awards of the Military Cross—Secretarial Posts for "The Unfit"—Sir R. Borden and Canadian War Hospitals—The Exhibition of Artificial Limbs—This Weeks' Drug Market—Charing Cross Hospital.

**Hospitals and the Spirit Tax** ... ... ... 371

Where the British Medical Association's Case Fails.

**Research and Remedies** ... ... ... ... 372

The Use of Salvarsanised Serum.

Radium in Gynæcology.

**Memorials to Medical Men** (*illustrated*) ... ... 373

I.—Westminster Abbey: Sir Peter Warren—Richard Mead—Hugo Chamberlen—Sir John Pringle—Sir James Young Simpson, Bart.

[*See over.*]

### CONTENTS—(*continued*).

PAGE

**Mental Hospitals and Recruiting** .. ... ... 375
Replacing Attendants by Women Nurses.
**Relieving Pain on the Battlefield** ... ... 375
**Round the World** ... ... ... ... ... 376
Fellow-Workers and the Roll of Honour.
**Hospital Architecture and Construction** ... 377
Derbyshire Royal Infirmary.
**Buildings Contemplated and in Progress** ... 377
**Gifts and Bequests** ... ... ... ... ... 377
**The School Doctor's Outfit** ... ... ... 378
I.—The Lesson of Experience.
**The Editor's Letter-Box** ... ... ... ... 379
Mental Hospitals and Recruiting.
The West London Hospital and the Three Examining Boards in London.
Dispensary Attendances.
Economy and Hospital Gardens.
The Hospital Egg.
**The Heating of Hospitals** ... ... ... ... 381
Leicester Infirmary: Heating by Steam Below Atmospheric Pressure.

PAGE

**Death of a Hospital Treasurer** ... ... ... 382
**Medical Appointments** ... ... ... ... vi
**A Policy of Parades** ... ... ... ... ... vi
**The Book Market** ... ... ... ... ... vi
**Latest Installations in Hospital Equipment** ... vi
What Others Are Doing.
**The Institutional Worker** ... ... (Suppl.) 1
**The Monthly Visitor—**
How to Obtain the Fullest Value from Surprise Visits.
Question for July.
Questions Invited.
Enquiries and Answers.
The Sick and In Need: Home for Case of Advanced Consumption—Maternity Case—Help for Inebriate Man—Women's Protection Societies—Institutions for Consumptives.
Miscellaneous: Massage Handbooks.
Employment and Training: Nursing in South America—Training in Laundry Work—List of Training Schools.

